# The Association Between Anastomotic Leak and 5-Year Oncologic Outcomes in Patients Undergoing Colorectal Cancer Resection: A Retrospective Cohort Study

**DOI:** 10.3390/curroncol33100587

**Published:** 2026-09-30

**Authors:** Ali Farsi, Mohammed Nassif

**Affiliations:** Department of Surgery, Faculty of Medicine, King Abdulaziz University, Jeddah 21589, Saudi Arabia; mnassif2@kau.edu.sa

**Keywords:** anastomotic leak, colorectal cancer, adenocarcinoma, oncologic outcomes

## Abstract

Background: Anastomotic leak (AL) is a serious complication following colorectal cancer (CRC) resection. Its influence on long-term oncologic outcomes, including recurrence and survival, remains controversial. Methods: This retrospective cohort study aimed to evaluate the prevalence of AL following CRC resection with anastomosis for stage 1–3 CRC. Clinical, operative, oncologic, and follow-up data were collected. AL was diagnosed within 30 days postoperatively. Primary outcomes included recurrence and mortality. Results: A total of 204 patients were included, of whom 25 (12.3%) developed AL. CRC recurrence occurred in 28.0% of patients with AL compared with 24.6% without AL (*p* = 0.712). Median time to recurrence was shorter in patients with AL (7 vs. 15 months, *p* = 0.064). One-year mortality was higher in patients with AL (20.0% vs. 3.9%, *p* = 0.008), and time to mortality was shorter (7 vs. 35 months, *p* = 0.048). However, AL was not independently associated with mortality or poor outcome on multivariable analysis. Conclusions: AL following CRC resection was associated with earlier mortality and a trend toward earlier recurrence, although no independent association with long-term oncologic outcomes was demonstrated. A larger prospective study with standardized leak definitions would better clarify the relationships between AL and CRC outcomes.

## Simple Summary

Colorectal cancer surgery often involves reconnecting the bowel after removing the tumor. Sometimes the bowel connection, called an anastomosis, breaks down and leaks intestinal contents into the abdomen. Previous studies have suggested that when this happens it may worsen long-term cancer outcomes, but results have been inconsistent. In this study, we evaluated whether anastomotic leak affected cancer recurrence and survival in patients undergoing colorectal cancer surgery. We found that patients who developed a leak had earlier recurrence and significantly higher risk of death within the first year after surgery. However, an anastomotic leak was not independently associated with worse long-term survival or recurrence after adjusting for other factors. Patients who required reoperation for severe leaks appeared to have poorer outcomes than those managed without surgery. These findings suggest that severe postoperative complications may influence early recovery and cancer progression, and highlight the need for larger prospective studies on this topic.

## 1. Introduction

Anastomotic leak (AL) is a potentially devastating complication that occurs after colorectal resection and anastomosis, occurring in about 3% to 20% of cases. Risk factors for AL include patient age and gender, along with the type of surgery and neoadjuvant radiotherapy, among many others. In addition to its immediate morbidity, the mortality rate associated with AL can vary in the range 0.8−27%. Aside from this, studies have shown that AL can have long-term oncologic implications for patients with colorectal cancer (CRC) [1,2].

Among patients with CRC, up to 25% of cases present initially with metastasis. Among those without metastasis at presentation, recurrence occurs in about 40% of patients and is associated with many risk factors, including tumor stage, differentiation, lymphovascular invasion, perineural invasion, tumor budding, resection margin status, and emergency presentation. An additional risk factor for cancer recurrence and survival that has been extensively studied is AL. When AL occurs, it is suspected to affect the body’s postoperative immune response, preserving the oncologic competence of viable tumor cells. However, the results of studies on this topic are conflicting. Some studies show that AL may worsen long-term outcomes; for example, a systematic review of 145 articles, including 154,981 patients, showed that AL was associated with an increased risk of CRC recurrence and decreased long-term survival. However, other studies have been less definitive, showing decreased survival but no effect on recurrence [1,3,4,5]. One systematic review and meta-analysis of 34 non-randomized studies including 78,434 patients reported that AL was associated with increased local recurrence and poorer overall, cancer-specific, and disease-free survival, while no significant association with distant recurrence was demonstrated [6]. Another meta-analysis focused solely on colon cancer and included 69,047 patients. It found impaired overall, disease-free, and cancer-specific survival after AL but no statistically significant increase in either local or distant recurrence [7]. For rectal cancer specifically, a meta-analysis of 18 cohort studies involving 34,487 patients found an association between AL and local recurrence and poorer long-term survival, but not distant recurrence [8]. These differences suggest that the oncologic impact of AL may vary according to tumor location, leak severity, study design, and the outcome assessed, and they underscore the need for studies that use consistent definitions and distinguish clinically significant leaks from minor radiologic findings. Fully understanding the effect of AL on recurrence and survival is important, as it can impact patient follow-up and the necessity of further adjuvant therapy.

In this study, we aimed to assess the prevalence of AL among patients undergoing resection for CRC and to assess its association with recurrence and survival.

## 2. Methodology

This study was conducted in accordance with the principles outlined in the Declaration of Helsinki (1975, revised in 2013). Approval to perform the study was obtained from the Unit of Biomedical Ethics at the Faculty of Medicine, King Abdulaziz University in Jeddah, Saudi Arabia (reference no. 143-26, obtained on 21 March 2026). The requirement for informed consent was waived by the Unit for Biomedical Ethics due to the retrospective non-interventional nature of this study.

A review of the hospital’s electronic medical records and operating room records was performed to identify all patients undergoing colorectal surgery from January 2013 to June 2020. Inclusion criteria were met by patients aged 18 to 110, who underwent a colorectal resection and anastomosis, and whose final pathology showed colorectal adenocarcinoma. Patients were excluded who underwent non-resectional surgery, who had no anastomosis, or who had surgery for benign disease. Also excluded were patients who did not have a pathologic specimen that showed adenocarcinoma, either on preoperative biopsy or final pathology. For the patients who met the inclusion criteria and none of the exclusion criteria, data were extracted related to their preoperative demographics, including age, gender, body mass index (BMI), baseline laboratory data, preoperative CT scans for evidence of metastasis, and need for neoadjuvant therapy. Their surgical records were reviewed for variables including American Society of Anesthesiology (ASA) score, operation approach and duration, need for transfusion, and urgency of the procedure, among others. The patient charts were then reviewed for postoperative course, duration of stay, follow-up, and survival. The charts were also evaluated for the use of chemotherapy after surgery, recording its administration at any point in time postoperatively. Histopathology was reviewed for pathologic stage, and only patients with stage 1, 2, and 3 based on final pathology were included. Patients with stage 4 at diagnosis or at the time of surgery were excluded. The patients’ medical records were reviewed for any surgery, CT imaging, or CT reports in the 30 postoperative days. AL was diagnosed under the following circumstances: if the patient was reoperated within 30 days of the index surgery and if there was bile, pus, or feces in the abdomen related to a disruption in the anastomosis; or if there was CT evidence of anastomotic leakage in the form of anastomotic disruption with perianastomotic fluid or air, contrast extravasation, or perianastomotic abscess within 5 cm of the anastomosis. Postoperative follow-up notes and imaging were reviewed for any evidence of CRC recurrence, either locally or distant; if it was found, the first date it was identified was recorded. Recurrence was also recorded in patients whose progress notes stated CRC diagnosis at an external institution. In this study, we evaluated only for the presence of recurrence, be it local or distant, without dividing patients by the location of recurrence. If the patient died at any point during follow-up, the date of death was recorded. Survival was counted from the day of surgery until the date of last follow-up or death.

### Data Analysis Plan

- Data were analyzed using IBM SPSS Statistics 27.0.
- Categorical variables are summarized as frequencies and percentages. Continuous variables are expressed as mean ± standard deviation and are compared between groups using the independent samples *t*-test.
- For the main outcome variables of the study, normality testing was performed using the Shapiro–Wilk test and visual inspection of histograms and Q–Q plots. Non-normally distributed continuous variables are expressed as median with interquartile range (IQR) and were compared using the Mann–Whitney U test.
- Associations between categorical variables were assessed using Pearson’s chi-square test. Fisher’s exact test was used for 2 × 2 tables when expected cell counts were small, and the Fisher–Freeman–Halton exact test was used for categorical variables with more than two categories and small expected cell counts.
- Time-to-event outcomes, including overall survival and recurrence-free survival, were evaluated using Kaplan–Meier survival analysis, and differences between the groups were compared using the log-rank test.
- Univariable and multivariable Cox proportional hazards regression analysis was performed to identify independent predictors of mortality and poor outcome, defined as recurrence and/or mortality. Results of Cox regression were reported as unadjusted and adjusted hazard ratios with 95% confidence intervals. A *p* value of <0.05 was considered statistically significant.
- To reduce the risk of overfitting, only variables with a univariable *p* value < 0.20 were considered for the multivariable Cox regression models. Anastomotic leak was included regardless of its univariable significance because it was the main exposure of interest. The final mortality model included 7 predictors for 52 deaths, giving approximately 7.4 events per predictor, whereas the poor-outcome model included 6 predictors for 73 events, giving approximately 12.2 events per predictor. This allowed the models to remain relatively parsimonious while still accounting for variables that were clinically relevant or showed a potential association with the outcomes.

## 3. Results

During the study period, 329 patients underwent operation for colorectal adenocarcinoma, of which 243 were stage 1, 2, and 3. Among these patients, 39 patients did not have an anastomosis and were excluded from the analysis. Therefore, a total of 204 patients who underwent CRC surgery with anastomosis were included in the analysis. Anastomotic leak was observed in 25 patients (12.3%), while 179 (87.7%) had no AL. The mean age of the cohort was 59.5 ± 12.7 years, with a range of 23 to 99 years, and most patients were male (61.8%). The mean preoperative serum albumin level was significantly lower among patients with AL compared with those without (30.4 ± 6.9 g/L vs. 34.0 ± 6.5 g/L; *p* = 0.012). Emergency surgery was also more frequent in the AL group (36.0% vs. 15.1%; *p* = 0.021). A history of previous venous thromboembolism (8.0% vs. 0.6%; *p* = 0.040) and cerebrovascular disease (12.0% vs. 1.7%; *p* = 0.026) was significantly more common among patients with AL. The type of surgery differed significantly between groups (*p* = 0.020), with low anterior resection being the most frequent procedure among patients with AL. Other baseline characteristics, including age, gender, ASA class, tumor stage, tumor site, diabetes, anemia, hypoalbuminemia as a categorical variable, chemotherapy, and radiotherapy among rectal tumor cases, were not significantly different between the two groups (Table 1).

Among the 25 patients who developed AL, 11 (44.0%) underwent reoperation, whereas 14 (56.0%) were managed without reoperation. The most frequent re-operative procedure was repair of the anastomosis with diversion, performed in 4 of 11 patients (36.4%), followed by irrigation and drainage with diversion and resection with re-anastomosis and diversion, each performed in 2 patients (18.2%). Other procedures included irrigation and drainage, resection with re-anastomosis, and transrectal drainage (Table 2).

The median (IQR) duration of follow-up for the whole cohort was 67 (36−85) months or 5.5 (3−7) years. Recurrence occurred in 51 patients (25.0%) during follow-up. The median time to recurrence was shorter among patients with AL compared with those without leak, although this did not reach statistical significance. The mean time to recurrence was 7 months (range of 3–24 months) in patients with AL vs. 15 months (range of 12–36 months) in those without AL (*p* = 0.064). Overall recurrence was observed in 28.0% of patients with AL and 24.6% of patients without AL (*p* = 0.712). Similarly, recurrence at 1 year, 3 years, and 5 years was numerically higher in the AL group, but the differences were not statistically significant (Table 3). During the follow-up period, mortality was reported in 52 patients (25.5%). Patients with AL had a significantly shorter median time to mortality compared with those without a leak (7 months, with a range of 4–32 months vs. 35 months, with a range of 19–62 months; *p* = 0.048). Although overall mortality was higher in the AL group than in the non-AL group (32.0% vs. 24.6%), this difference was not statistically significant (*p* = 0.425). One-year mortality was significantly higher among patients with AL (20.0% vs. 3.9%; *p* = 0.008). However, mortality at 3 years and 5 years did not differ significantly between the two groups (Table 4).

Outcomes were also compared between cases who underwent reoperation and all others, including cases with AL managed conservatively and cases without AL. Cases who underwent reoperation had a significantly shorter median time to CRC recurrence than those who did not undergo reoperation (5 months, with a range of 3–13] months vs. 15 months, with a range of 11–35 months; *p* = 0.022). Overall recurrence was higher in the reoperation group (36.4% vs. 24.4%), but the difference was not statistically significant (*p* = 0.472). Recurrence at 1, 3, and 5 years also did not differ significantly between the groups (all *p* > 0.05) (Table 5). Overall mortality was also numerically higher among patients who underwent reoperation (45.5% vs. 24.4%), but this difference was not statistically significant (*p* = 0.152). One-year mortality was significantly higher among patients who underwent reoperation (27.3% vs. 4.7%; *p* = 0.020), 3-year mortality showed a borderline difference (36.4% vs. 13.5%; *p* = 0.060), whereas 5-year mortality did not differ significantly between groups (*p* = 0.231) (Table 6).

**Figure 1:**
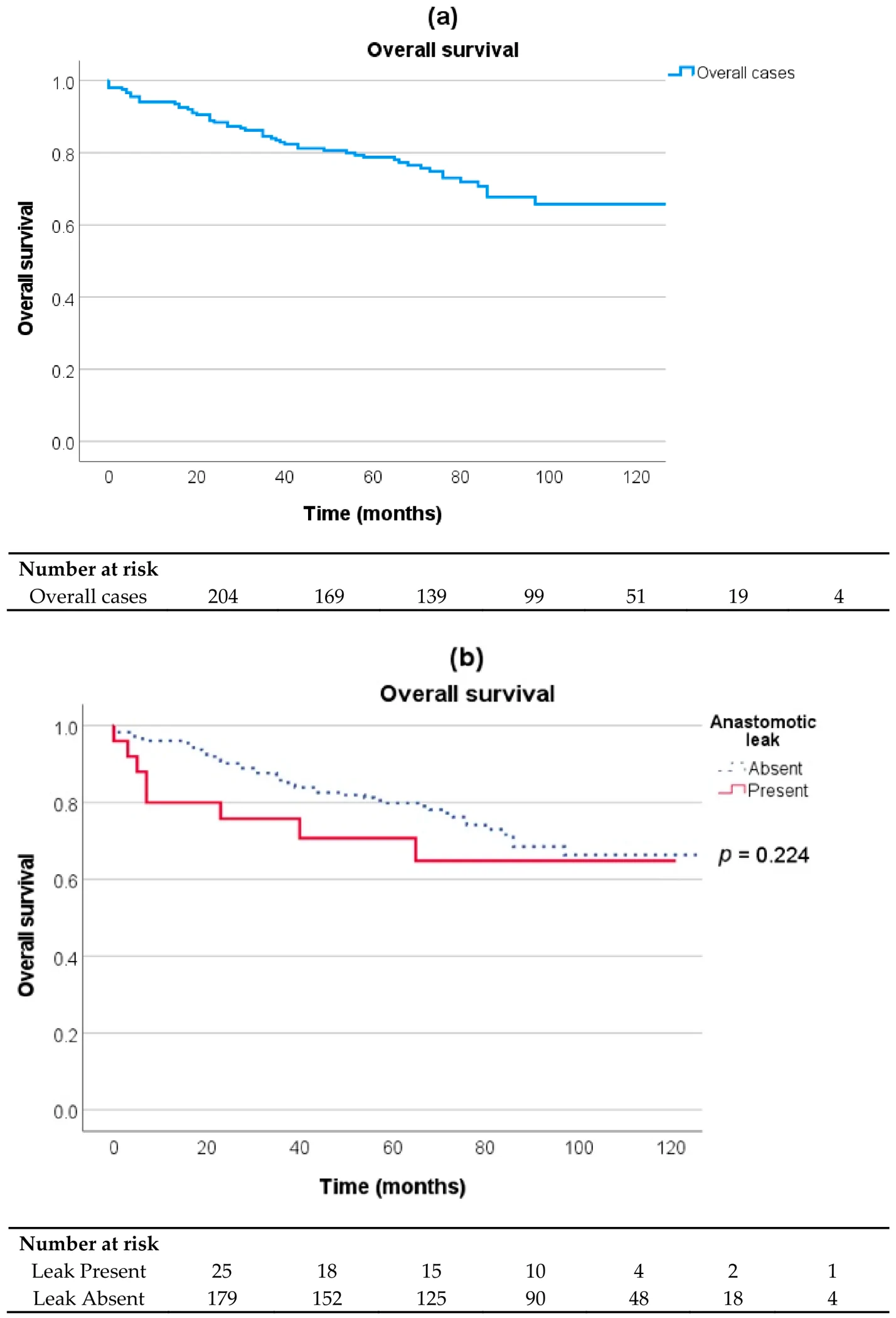
Kaplan–Meier graphs showing overall survival in (**a**) all cases and (**b**) cases with and without anastomotic leak.

**Figure 2:**
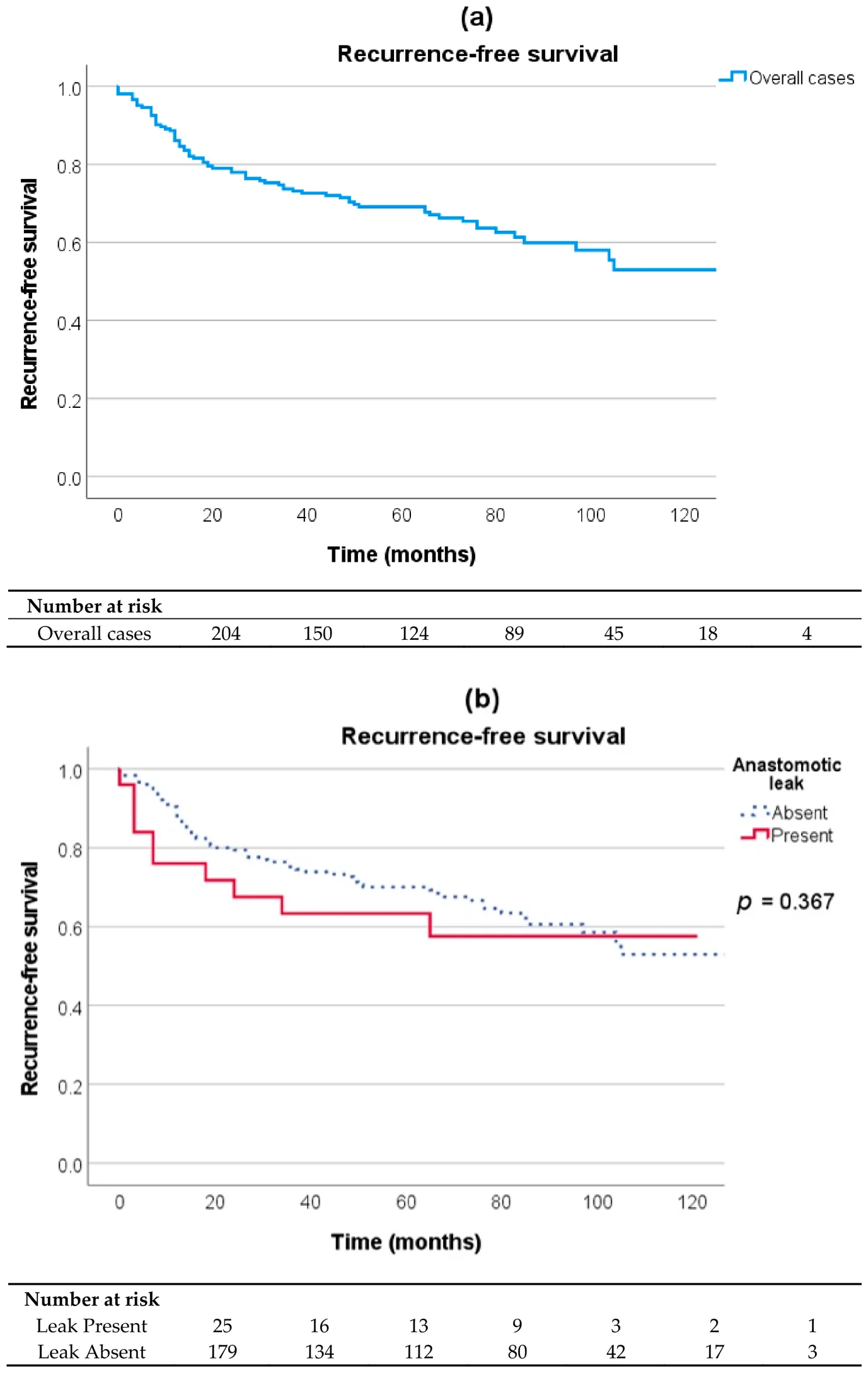
Kaplan–Meier graphs showing recurrence-free survival in (**a**) all cases and (**b**) cases with and without anastomotic leak.

Kaplan–Meier analysis demonstrated a gradual decline in overall survival over time in the entire cohort. When stratified by AL status, patients with leak showed lower early survival compared with those without leak; however, the difference in overall survival between the two groups was not statistically significant on log-rank testing (*p* = 0.224) (Figure 1a,b). Similarly, recurrence-free survival declined over the follow-up period, and patients with AL showed an earlier decline in recurrence-free survival. However, the difference between AL and non-AL groups was not statistically significant (*p* = 0.367) (Figure 2a,b).

In univariable Cox proportional hazards regression analysis for mortality, age > 60 years, ASA class III–IV, emergency surgery, stage III disease, and hypoalbuminemia were significantly associated with increased mortality risk. In the multivariable model, stage III disease remained the strongest independent predictor of mortality, with more than threefold higher risk compared with stage I–II disease (aHR: 3.28, 95% CI: 1.81–5.93; *p* < 0.001). Age > 60 years was also independently associated with increased mortality risk (aHR: 2.49, 95% CI: 1.32–4.70; *p* = 0.005). ASA class III–IV, emergency surgery, diabetes, and hypoalbuminemia were not independently associated with mortality after adjustment. Anastomotic leak was also not independently associated with mortality (aHR: 1.56, 95% CI: 0.71–3.44; *p* = 0.270). The overall multivariable Cox model was statistically significant (score χ^2^ = 43.916, df = 7, *p* < 0.001) (Table 7).

For poor outcome, defined as recurrence and/or mortality, univariable Cox regression showed significant associations with age > 60 years, ASA class III–IV, emergency surgery, and stage III disease. In the multivariable model, stage III disease remained the strongest independent predictor of poor outcome (aHR: 3.36, 95% CI: 2.05–5.51; *p* < 0.001). Age > 60 years was also independently associated with an increased risk of poor outcome (aHR: 1.94, 95% CI: 1.18–3.19; *p* = 0.009). ASA class III–IV and emergency surgery showed higher adjusted hazard estimates but did not reach statistical significance (*p* = 0.080 and *p* = 0.075, respectively). Hypoalbuminemia was not independently associated with poor outcome, and anastomotic leak also remained non-significant after adjustment (aHR: 1.28, 95% CI: 0.64–2.54; *p* = 0.486). The overall multivariable Cox model was statistically significant (score χ^2^ = 45.433, df = 6, *p* < 0.001) (Table 8).

Number of events: 52

HR: hazard ratio; CI: confidence interval. Bold values indicate statistical significance at *p* ≤ 0.05. Variables with *p* < 0.20 on univariable analysis were entered into the multivariable model; anastomotic leak was retained irrespective of its univariable *p* value, because it was the primary exposure of interest. The univariable estimates are taken from the individual Cox models.

Model fit information**:** The multivariable Cox proportional hazards model was statistically significant overall (score χ^2^ = 43.916, df = 7, *p* < 0.001). The final −2 log likelihood was 472.544.

Number of events: 73

HR: hazard ratio; CI: confidence interval. Bold values indicate statistical significance at *p* ≤ 0.05. Variables with *p* < 0.20 on univariable analysis were entered into the multivariable model; anastomotic leak was retained irrespective of its univariable *p* value because it was the primary exposure of interest. The univariable results show significant associations for age, ASA class, emergency surgery, and tumor stage.

Model fit information: The multivariable Cox proportional hazards model was statistically significant overall (score χ^2^ = 45.433, df = 6, *p* < 0.001). The final −2 log likelihood was 676.608.

## 4. Discussion

In this study, we aimed to evaluate the proportion of patients undergoing colorectal resection and anastomosis who develop an anastomotic leak, and to assess the association between AL and overall survival and recurrence in patients with CRC.

We found that AL occurred in 12.3% of patients, which is within the range of 3−20% previously reported in the literature [1]. In line with previous research, patient baseline characteristics revealed an association between AL status and urgency of the procedure, albumin level, and type of procedure [2]. Cerebrovascular disease and venous thromboembolism were also significantly associated with AL status, but the numbers of patients were small.

In our study, median time to CRC recurrence was shorter in patients with AL, but this did not reach statistical significance. Recurrence rate was also slightly higher in patients with AL, but this also failed to reach statistical significance, in line with results from other studies [9]. Accordingly, these findings should not be interpreted as evidence that AL increased recurrence risk in our cohort. The numerical differences in recurrence may reflect limited statistical power related to the small number of patients with AL and should therefore be considered hypothesis-generating. When patients were categorized into those with AL treated surgically and those who did not have AL or had AL treated non-surgically, a statistically significant difference in median time to recurrence was identified (5 vs. 15 months, respectively). There are several potential explanations for this.

First, the combined physiologic insult from a significant AL and reoperation (with accompanying general anesthesia, which some studies have suggested may have its own immunosuppressive effect), leads to a significant systemic inflammatory response, which may enhance tumor recurrence from residual cancer cells and dormant micrometastasis. Additionally, physiologic insult from postoperative sepsis and reoperation may lead to a delay in chemotherapy. Postoperative chemotherapy presents the greatest benefit when initiated within 6 to 8 weeks after resection, when it can lead to a 50% reduction in disease recurrence [1,9,10,11]; initiation after this period may decrease the oncologic benefit of adjuvant therapy. On multivariate analysis, we found that patients with stage 3 CRC had a reduced recurrence-free survival. In addition to the increased recurrence rate associated with the higher stage, these patients may have suffered a delay in chemotherapy if they developed an AL (though in our study, we did not assess the duration from surgery to the start of adjuvant chemotherapy).

Heterogeneity in study results concerning the effect of AL on recurrence can also be attributed to the various methods of assessment for AL (whether clinical, radiologic, or endoscopic), the severity of AL, and the varying definitions of AL. For example, one study has defined AL only in terms of anastomotic dehiscence at reoperation or contrast extravasation on imaging; perianastomotic fluid was not considered symptomatic of AL, instead it was considered a surgical site infection (SSI) [5]. In another example, a large study of 88,154 CRC patients from the Netherlands cancer registry only included patients with AL who were readmitted or required re-intervention, potentially missing those who had a small contained leak managed without surgery or radiologic drainage during the initial admission; this study found that AL worsened survival but did not independently influence recurrence [1]. Taking the severity of AL into account is important, as one study from Japan showed that patients with AL who underwent surgical treatment had poorer long-term outcomes than those treated non-surgically [11].

Another potential reason for the disparity in recurrence results is that many studies combine colon and rectal cancer patients, and the influence of AL on recurrence may vary in these patients. Indeed, in a study based on data from the Colorectal Cancer Laparoscopic or Open Resection (COLOR and COLOR II) trials, AL in the setting of rectal anastomosis was found to influence local recurrence and disease-free survival, while AL in the setting of colonic anastomosis did not [1,12,13]. Large meta-analysis also demonstrated this disparity. Ha et al. reported increased local recurrence and reduced overall, cancer-specific, and disease-free survival after AL in a meta-analysis of colorectal cancer surgery,[6] whereas Mohamed et al., analyzing colonic cancer separately, found worse survival outcomes without a significant increase in recurrence [7]. Meta-analytic data in rectal cancer have more consistently demonstrated an association with local recurrence and reduced survival [8]. As previously mentioned, interpretation across studies is complicated by substantial variation in how AL is defined, diagnosed, graded, and reported. A 2024 systematic review of colorectal cancer trials found marked inconsistency in AL definitions and severity grading, supporting the need for standardized reporting in future outcome studies [14]. When studies have shown an association between AL and recurrence, the authors suggest that tumor cells that can be found in the colonic lumen and on stapling devices disseminate when AL occurs. Along with the altered systemic inflammation that accompanies AL, this can alter humoral and cell-mediated immunity, enhancing micrometastasis [1,4,9,10,12].

With regard to mortality in our patient population, we found that time to mortality was significantly shorter in patients with AL compared to those without (7 vs. 35 months, respectively), and that one-year mortality was significantly greater in patients with AL. However, this difference in mortality disappeared during the follow-up period. Though studies have shown varying association between AL and recurrence, most have shown an increased mortality in patients with AL. When decreased survival occurs without an increase in recurrence, authors suggest physiologic insult from AL, leading to increased frailty and the worsening of pre-existing co-morbidities [1,9].

Though we did not include patients with stage 4 disease, the role of palliative resection of tumors in stage 4 CRC patients remains controversial. However, while results are conflicting on whether it improves survival, stage 4 patients with AL have been shown to suffer poor outcomes, with a significant decrease in survival, probably related to significant disease progression secondary to clinical deterioration and sepsis-related immunosuppression. This presents the surgeon with a difficult decision: namely, whether he should anastomose a patient at high risk of AL (if even possible), given the higher risk of disease progression if AL unfortunately occurs [1].

An aspect of surgical management that has recently gained attention regarding its impact on cancer recurrence is bowel preparation. While we did not assess for it, bowel preparation has been suggested as a means to decrease the fecal and bacterial load preoperatively, thereby decreasing the risk of AL. A recent retrospective cohort study of 1279 patients found improved 5-year disease-free survival in patients who underwent a preoperative mechanical and oral antibiotic bowel preparation compared to those who did not [5]. Though the exact mechanism is not understood, the role of the microbiome in carcinogenesis and cancer recurrence is increasingly recognized and has been proposed as a possible cause. This is thought to be mediated through multiple pathways. For example, murine models suggest a role for collagenase-producing bacteria like *Enterococcus* and *Proteus* species (the most commonly isolated bacteria in patients who develop AL); these organisms are thought to activate components of the extracellular matrix that promote tumorigenesis [5,15]. It has also been suggested that the colonic bacteria present perioperatively can influence the ability of exfoliated tumor cells to reimplant and cause postoperative tumor recurrence [5,15]. Therefore, some authors propose that by altering the perioperative microbiome, either with the use of bowel preparation, oral antibiotics, prebiotics, probiotics, or synbiotics, it may be possible to improve cancer outcomes [5,15].

Our study has several limitations. First, it is a retrospective study, which presents challenges concerning the collection of variables. Some of the patients continued their follow-up at external institutions, which could have influenced the number of recurrences identified. Second, the study included a relatively small number of patients and a small number of events. The observed numerical differences in recurrence and overall survival did not reach statistical significance and should not be interpreted as evidence of an adverse oncologic effect of AL. The limited number of AL cases and outcome events may have reduced statistical power, and larger, adequately powered cohorts are needed to determine whether a true association exists. Third, both colon and rectal cancer patients were included in our study; the effect of AL on recurrence and mortality may differ for these cancers, as has been shown in previous studies [10]. Fourth, differences in baseline characteristics were identified between patients with AL and those without. The strengths of our study include the relatively long median follow-up of 5.5 years; the assessment of both recurrence and survival; inclusion of a large group of important clinical and oncological variables; the finding that the AL rate was within previously reported ranges; and our separate assessment of the impact of severe AL.

## 5. Conclusions

In this retrospective cohort of patients undergoing CRC resection with anastomosis, AL was associated with significantly higher 1-year mortality and a shorter time to mortality. Although patients with AL had a numerically shorter time to recurrence and higher recurrence rates, these recurrence differences were not statistically significant. Furthermore, AL was not independently associated with mortality or poor outcome. These findings therefore do not establish AL as an independent determinant of long-term oncologic outcome and should be interpreted in the context of the retrospective single-center design and the small number of AL cases and outcome events. Clinically, AL—particularly a severe leak requiring reoperation—may identify a vulnerable postoperative group with increased early morbidity and mortality, but our data do not support altering oncologic treatment or surveillance solely on the basis of AL. Future prospective multicenter studies should include larger cohorts, standardized definitions and severity grading of AL, detailed assessment of timing and delivery of adjuvant therapy, and adequate adjustment for tumor stage and other oncologic prognostic factors.

## Figures and Tables

**Table 1:** Baseline characteristics of patients undergoing colorectal cancer surgery overall and according to anastomotic leakage status (N = 204).

Characteristic	CRC Surgery Overall (*n* = 204)	AL Present (*n* = 25)	AL Absent (*n* = 179)	*p* Value
Continuous variables, mean ± SD				
Age, years	59.5 ± 12.7	60.6 ± 14.6	59.4 ± 12.4	0.646 β
Preoperative serum creatinine, µmol/L	75.0 ± 26.7	69.4 ± 20.1	75.8 ± 27.4	0.264 β
Preoperative serum albumin, g/L	33.5 ± 6.6	30.4 ± 6.9	34.0 ± 6.5	0.012 β
Hemoglobin, g/dL	11.4 ± 2.0	11.3 ± 1.8	11.4 ± 2.0	0.780 β
Categorical variables, *n* (%)				
Gender				0.846 α
Male	126 (61.8)	15 (60.0)	111 (62.0)	
Female	78 (38.2)	10 (40.0)	68 (38.0)	
Urgency				0.021 †
Routine	168 (82.4)	16 (64.0)	152 (84.9)	
Emergency	36 (17.6)	9 (36.0)	27 (15.1)	
ASA class				0.066 γ
I	9 (4.4)	2 (8.0)	7 (3.9)	
II	125 (61.3)	11 (44.0)	114 (63.7)	
III	65 (31.9)	10 (40.0)	55 (30.7)	
IV	5 (2.5)	2 (8.0)	3 (1.7)	
Primary tumor site				0.072 γ
Ascending colon	22 (10.8)	0 (0.0)	22 (12.3)	
Cecum	25 (12.3)	4 (16.0)	21 (11.7)	
Descending colon	17 (8.3)	3 (12.0)	14 (7.8)	
Rectosigmoid	31 (15.2)	3 (12.0)	28 (15.6)	
Rectum	42 (20.6)	8 (32.0)	34 (19.0)	
Sigmoid colon	51 (25.0)	3 (12.0)	48 (26.8)	
Transverse colon	16 (7.8)	4 (16.0)	12 (6.7)	
Tumor stage				0.267 α
1	51 (25.0)	9 (36.0)	42 (23.5)	
2	65 (31.9)	5 (20.0)	60 (33.5)	
3	88 (43.1)	11 (44.0)	77 (43.0)	
Type of surgery				0.020 γ
Right hemicolectomy	32 (15.7)	5 (20.0)	27 (15.1)	
Extended right hemicolectomy	8 (3.9)	0 (0.0)	8 (4.5)	
Transverse colectomy	4 (2.0)	3 (12.0)	1 (0.6)	
Left hemicolectomy	15 (7.4)	1 (4.0)	14 (7.8)	
Sigmoidectomy	49 (24.0)	2 (8.0)	47 (26.3)	
Subtotal colectomy	20 (9.8)	2 (8.0)	18 (10.1)	
Low anterior resection	72 (35.3)	12 (48.0)	60 (33.5)	
Others	4 (2.0)	0 (0.0)	4 (2.2)	
Diversion				0.286 α
Done	48 (23.5)	8 (32.0)	40 (22.3)	
Not done	156 (76.5)	17 (68.0)	139 (77.7)	
Diabetes				0.562 α
Present	76 (37.3)	8 (32.0)	68 (38.0)	
Absent	128 (62.7)	17 (68.0)	111 (62.0)	
Ischemic heart disease				0.279 †
Present	20 (9.8)	4 (16.0)	16 (8.9)	
Absent	184 (90.2)	21 (84.0)	163 (91.1)	
Heart failure				0.206 †
Present	7 (3.4)	2 (8.0)	5 (2.8)	
Absent	197 (96.6)	23 (92.0)	174 (97.2)	
Chronic liver disease				1.000 †
Present	5 (2.5)	0 (0.0)	5 (2.8)	
Absent	199 (97.5)	25 (100.0)	174 (97.2)	
Chronic kidney disease				0.353 †
Present	10 (4.9)	2 (8.0)	8 (4.5)	
Absent	194 (95.1)	23 (92.0)	171 (95.5)	
History of venous thromboembolism				0.040 †
Present	3 (1.5)	2 (8.0)	1 (0.6)	
Absent	201 (98.5)	23 (92.0)	178 (99.4)	
Cerebrovascular disease				0.026 †
Present	6 (2.9)	3 (12.0)	3 (1.7)	
Absent	198 (97.1)	22 (88.0)	176 (98.3)	
Hypoalbuminemia				0.114 α
Present	117 (57.4)	18 (72.0)	99 (55.3)	
Absent	87 (42.6)	7 (28.0)	80 (44.7)	
Anemia				0.852 α
Present	142 (69.6)	17 (68.0)	125 (69.8)	
Absent	62 (30.4)	8 (32.0)	54 (30.2)	
Radiotherapy for rectal tumors (*n* = 73)				1.000 †
Given	39 (53.4)	6 (54.5)	33 (53.2)	
Not given	34 (46.6)	5 (45.5)	29 (46.8)	
Chemotherapy				0.556 α
Given	103 (50.5)	14 (56.0)	89 (49.7)	
Not given	101 (49.5)	11 (44.0)	90 (50.3)	
Preoperative chemotherapy				0.171 †
Given	23 (11.3)	5 (20.0)	18 (10.1)	
Not given	181 (88.7)	20 (80.0)	161 (89.9)	
Postoperative chemotherapy				0.968 α
Given	89 (43.6)	11 (44.0)	78 (43.6)	
Not given	115 (56.4)	14 (56.0)	101 (56.4)	

Statistical tests for categorical variables: α Pearson’s chi-square test; † Fisher’s exact test; γ Fisher–Freeman–Halton exact test. Statistical tests for continuous variables: β Independent samples *t*-test. Statistical significance at *p* < 0.05. For radiotherapy, only rectal tumor cases were included (*n* = 73). Exact test outputs were used where the expected cell count was less than 5.

**Table 2:** Surgical management of patients with anastomotic leak (*n* = 25).

Characteristic	*n* (%)
Reoperation	
Done	11 (44.0)
Not done	14 (56.0)
Type of reoperation (*n* = 11)	
Repair of anastomosis with diversion	4 (36.4)
Irrigation and drainage	1 (9.1)
Irrigation and drainage with diversion	2 (18.2)
Resection and re-anastomosis with diversion	2 (18.2)
Resection and re-anastomosis	1 (9.1)
Transrectal drainage	1 (9.1)

Note: data are presented as *n* (%). Percentages for reoperation were calculated among patients with anastomotic leak only (*n* = 25). Percentages for type of reoperation were calculated among patients who underwent reoperation only (*n* = 11).

**Table 3:** CRC recurrence outcomes according to anastomotic leak status (N = 204).

Characteristic	Overall (*n* = 204)	AL Present (*n* = 25)	AL Absent (*n* = 179)	*p* Value
Time to recurrence (months), median (IQR)	15 (9–34)	7 (3–24)	15 (12–36)	0.064 β
Overall recurrence, *n* (%)				0.712 α
Present	51 (25.0)	7 (28.0)	44 (24.6)	
Absent	153 (75.0)	18 (72.0)	135 (75.4)	
1-year recurrence, *n* (%)				0.279 †
Present	20 (9.8)	4 (16.0)	16 (8.9)	
Absent	184 (90.2)	21 (84.0)	163 (91.1)	
3-year recurrence, *n* (%)				0.283 †
Present	40 (19.6)	7 (28.0)	33 (18.4)	
Absent	164 (80.4)	18 (72.0)	146 (81.6)	
5-year recurrence, *n* (%)				0.574 α
Present	48 (23.5)	7 (28.0)	41 (22.9)	
Absent	156 (76.5)	18 (72.0)	138 (77.1)	

Statistical tests for categorical variables: α Pearson’s chi-square test; † Fisher’s exact test. Statistical tests for continuous variables: β Mann–Whitney U test. Statistical significance at *p* < 0.05.

**Table 4:** Mortality outcomes according to anastomotic leak status (N = 204).

Characteristic	Overall (*n* = 204)	AL Present (*n* = 25)	AL Absent (*n* = 179)	*p* Value
Time to mortality (months), median (IQR)	31 (16–57)	7 (4–32)	35 (19–62)	0.048 β
Overall mortality, *n* (%)				0.425 α
Present	52 (25.5)	8 (32.0)	44 (24.6)	
Absent	152 (74.5)	17 (68.0)	135 (75.4)	
1-year mortality, *n* (%)				0.008 †
Present	12 (5.9)	5 (20.0)	7 (3.9)	
Absent	192 (94.1)	20 (80.0)	172 (96.1)	
3-year mortality, *n* (%)				0.222 †
Present	30 (14.7)	6 (24.0)	24 (13.4)	
Absent	174 (85.3)	19 (76.0)	155 (86.6)	
5-year mortality, *n* (%)				0.283 †
Present	40 (19.6)	7 (28.0)	33 (18.4)	
Absent	164 (80.4)	18 (72.0)	146 (81.6)	

Statistical tests for categorical variables: α Pearson’s chi-square test; † Fisher’s exact test. Statistical tests for continuous variables: β Mann–Whitney U test. Statistical significance at *p* < 0.05 was assumed.

**Table 5:** CRC recurrence outcomes according to reoperation status (N = 204).

Characteristic	Overall (*n* = 204)	Reoperated (*n* = 11)	Not Reoperated (*n* = 193)	*p* Value
Time to recurrence (months), median (IQR)	15 (9–34)	5 (3–13)	15 (11–35)	0.022 β
Overall recurrence, *n* (%)				0.472 †
Present	51 (25.0)	4 (36.4)	47 (24.4)	
Absent	153 (75.0)	7 (63.6)	146 (75.6)	
1-year recurrence, *n* (%)				0.080 †
Present	20 (9.8)	3 (27.3)	17 (8.8)	
Absent	184 (90.2)	8 (72.7)	176 (91.2)	
3-year recurrence, *n* (%)				0.231 †
Present	40 (19.6)	4 (36.4)	36 (18.7)	
Absent	164 (80.4)	7 (63.6)	157 (81.3)	
5-year recurrence, *n* (%)				0.291 †
Present	48 (23.5)	4 (36.4)	44 (22.8)	
Absent	156 (76.5)	7 (63.6)	149 (77.2)	

Statistical tests for categorical variables: † Fisher’s exact test. Statistical tests for continuous variables: β Mann–Whitney U test. Statistical significance at *p* < 0.05.

**Table 6:** Mortality outcomes according to reoperation status (N = 204).

Characteristic	Overall (*n* = 204)	Reoperated (*n* = 11)	Not Reoperated (*n* = 193)	*p* Value
Time to mortality (months), median (IQR)	31 (16–57)	7 (7–23)	35 (16–58)	0.222 β
Overall mortality, *n* (%)				0.152 †
Present	52 (25.5)	5 (45.5)	47 (24.4)	
Absent	152 (74.5)	6 (54.5)	146 (75.6)	
1-year mortality, *n* (%)				0.020 †
Present	12 (5.9)	3 (27.3)	9 (4.7)	
Absent	192 (94.1)	8 (72.7)	184 (95.3)	
3-year mortality, *n* (%)				0.060 †
Present	30 (14.7)	4 (36.4)	26 (13.5)	
Absent	174 (85.3)	7 (63.6)	167 (86.5)	
5-year mortality, *n* (%)				0.231 †
Present	40 (19.6)	4 (36.4)	36 (18.7)	
Absent	164 (80.4)	7 (63.6)	157 (81.3)	

Statistical tests for categorical variables: † Fisher’s exact test. Statistical tests for continuous variables: β Mann–Whitney U test. Statistical significance at *p* < 0.05 was assumed.

**Table 7:** Univariable and multivariable Cox proportional hazards regression analysis for mortality (N = 204).

Variable	Crude HR (95% CI)	*p* Value	Adjusted HR (95% CI)	*p* Value
Age ≥ 60 years	2.51 (1.40–4.49)	0.002	2.49 (1.32–4.70)	0.005
Male gender	0.89 (0.51–1.54)	0.664	—	—
ASA class (III–IV)	2.12 (1.23–3.65)	0.007	1.53 (0.87–2.68)	0.137
Stage (stage III vs. stage I–II)	2.83 (1.61–4.98)	<0.001	3.28 (1.81–5.93)	<0.001
Diabetes	1.49 (0.86–2.56)	0.156	1.18 (0.66–2.10)	0.580
Anemia	1.12 (0.61–2.04)	0.715	—	—
Hypoalbuminemia	2.24 (1.21–4.14)	0.010	1.76 (0.93–3.34)	0.085
Emergency surgery	2.24 (1.19–4.21)	0.012	1.62 (0.83–3.16)	0.161
Rectal tumor	0.68 (0.38–1.25)	0.215	—	—
Diversion colostomy/ileostomy	1.19 (0.64–2.20)	0.580	—	—
Anastomotic leak	1.59 (0.75–3.38)	0.229	1.56 (0.71–3.44)	0.270
Chemotherapy given	0.89 (0.51–1.53)	0.660	—	—

**Table 8:** Univariable and multivariable Cox proportional hazards regression analysis for poor outcome (recurrence and/or mortality) (N = 204).

Variable	Crude HR (95% CI)	*p* Value	Adjusted HR (95% CI)	*p* Value
Age > 60 years	1.71 (1.07–2.73)	0.025	1.94 (1.18–3.19)	0.009
Male gender	1.16 (0.72–1.88)	0.541	—	—
ASA class (III–IV)	1.93 (1.22–3.06)	0.005	1.52 (0.95–2.44)	0.080
Stage (stage III vs. stage I–II)	3.16 (1.95–5.11)	<0.001	3.36 (2.05–5.51)	<0.001
Diabetes	1.17 (0.73–1.86)	0.512	—	—
Anemia	1.19 (0.71–2.00)	0.503	—	—
Hypoalbuminemia	1.42 (0.88–2.28)	0.153	1.22 (0.74–2.01)	0.427
Emergency surgery	2.02 (1.17–3.49)	0.011	1.68 (0.95–2.96)	0.075
Rectal tumor	0.75 (0.46–1.23)	0.258	—	—
Diversion colostomy/ileostomy	0.88 (0.51–1.54)	0.663	—	—
Anastomotic leak	1.36 (0.70–2.65)	0.371	1.28 (0.64–2.54)	0.486
Chemotherapy given	1.06 (0.67–1.68)	0.803	—	—

## Data Availability

The data are restricted by the institutional ethics committee to protect participant privacy. De-identified data requests may be sent to the communicating author and maybe shared pending approval from the Unit of Biomedical Ethics in the Faculty of Medicine at King Abdulaziz University.
